# White Paper on Nutrition Sensing and Ageing

**DOI:** 10.1111/nbu.70020

**Published:** 2025-07-09

**Authors:** Aygul Dagbasi, Adrian Holliday, Bernadette Carroll, Chiara de Lucia, Oliver M. Shannon, John Mathers, John McLaughlin, Chloe French, Aylin Hanyaloglu, Sorrel Burden, Douglas Morrison, Viktor Korolchuk, Gary Frost

**Affiliations:** ^1^ Section of Nutrition, Department of Metabolism Digestion and Reproduction Faculty of Medicine, Imperial College London London UK; ^2^ School of Biomedical, Nutritional, and Sport Science Newcastle University Newcastle Upon Tyne UK; ^3^ Human Nutrition and Exercise Research Centre Population Health Sciences Institute, Newcastle University Newcastle Upon Tyne UK; ^4^ School of Biochemistry University of Bristol, University Walk Bristol UK; ^5^ Department of Basic and Clinical Neuroscience Institute of Psychiatry, Psychology & Neuroscience (CDL), King's College London London UK; ^6^ Centre for Age‐Related Medicine Stavanger University Hospital Stavanger Norway; ^7^ Division of Diabetes, Endocrinology and Gastroenterology, Faculty of Biology, Medicine and Health The University of Manchester & Northern Care Alliance NHS Foundation Trust, Salford Care Organisation Manchester UK; ^8^ Healthy Ageing Research Group School of Health Sciences, University of Manchester Manchester UK; ^9^ Department of Metabolism Digestion and Reproduction, Faculty of Medicine Imperial College London London UK; ^10^ Scottish Universities Environmental Research Centre (SUERC), University of Glasgow East Kilbride UK; ^11^ Biosciences Institute, Faculty of Medical Sciences Newcastle University Newcastle upon Tyne UK

**Keywords:** ageing, healthy ageing, network, nutrition, nutrition sensing

## Abstract

Ageing, which is defined as the progressive deterioration of physiological functions, is an inevitable part of the lifecycle. Nevertheless, its progress is believed to be influenced by modifiable factors, one of the most important being dietary intake. Like many other systems within the human body, detection of nutrients (defined as nutrition sensing), their metabolism, and the body's response to nutrients may change with ageing. There is compelling evidence to suggest that nutrition sensing mechanisms can become dysregulated in certain ageing adults, which can lead to increased morbidity and mortality. However, there is still much to unravel in nutrition sensing and its impact on ageing on multiple levels from molecular signalling to the food environment. We hypothesise that nutrition sensing mechanisms play an important role in the ageing process. To this end, we formed the Ageing and Nutrition Sensing Network to bring together leading multi‐disciplinary researchers and early career researchers with expertise across ageing, cell biology, nutrition, epidemiology, and policy. The network aims to address the priority area of health span and quality of life in older age. As a consortium, we defined nutrition sensing and identified five key challenges to be addressed to advance the field of nutrition sensing and ageing. This resulted in the development of four main projects, each one embracing multidisciplinary working and investigating nutrition sensing and ageing from different perspectives. Here we describe our network, our projects, and how we plan to incorporate our findings to promote healthy ageing from science and industry to policy.

## 1 Introduction

Ageing is an inevitable and progressive process characterised by the accumulation of functional deficits across multiple organ systems (Lowsky et al. 2013; López‐Otín et al. 2013; WHO 2024). These functional deficits reduce mental and physical capabilities and drive the development of common non‐communicable diseases including cardiovascular disease, cancers, and neurodegenerative diseases (Niccoli and Partridge 2012) which are major contributors to morbidity and to premature mortality globally. Despite such inevitability, the world population is living longer than before. The number of people aged 80 years and over is predicted to triple by 2050 (WHO 2024). Therefore, it has become crucial to understand how health can be maintained better during ageing.

The ageing trajectory is influenced by modifiable behaviours, notably diet and physical activity in addition to the non‐modifiable factors such as the genotype. Diet dictates energy and nutrient availability, which have profound effects on the functionality of body systems during ageing. For example, although only confirmed using biomarkers of ageing, rather than extended longevity in humans, studies in model organisms have shown that dietary (energy) restriction is one of the most effective interventions for extending lifespan and for maintaining better health during ageing (Fontana 2006; Waziry et al. 2023). Indeed, nutrient intakes and availabilities along with dietary patterns are associated with age‐related health outcomes, morbidity, and mortality in humans (Micha et al. 2017; Dahl and Stewart 2015; Shannon et al. 2021, 2023; Wickramasinghe et al. 2020; Malcomson and Mathers 2019).

However, the body's response to nutrients, and the body's requirements for nutrients change across the life course (Holliday et al. 2025; Dorrington et al. 2020; Fujita et al. 2009). Accordingly, deregulated nutrition sensing is one of the twelve hallmarks of ageing that are seen in all ageing organisms (López‐Otín et al. 2023). In later life, the body may develop resistance to some nutrients, such as the anabolic resistance to amino acids (Fujita et al. 2009). Some systems, across multiple organs and tissues, responsible for detecting and metabolising nutrients, such as the appetite regulating hormones, may become dysregulated (Dagbasi, Warner, et al. 2024). There is also compelling evidence from animal models that gene‐nutrient interaction influences the ageing trajectory through epigenetic modifications (Dato et al. 2016; Westendorp 2006).

**FIGURE 1:**
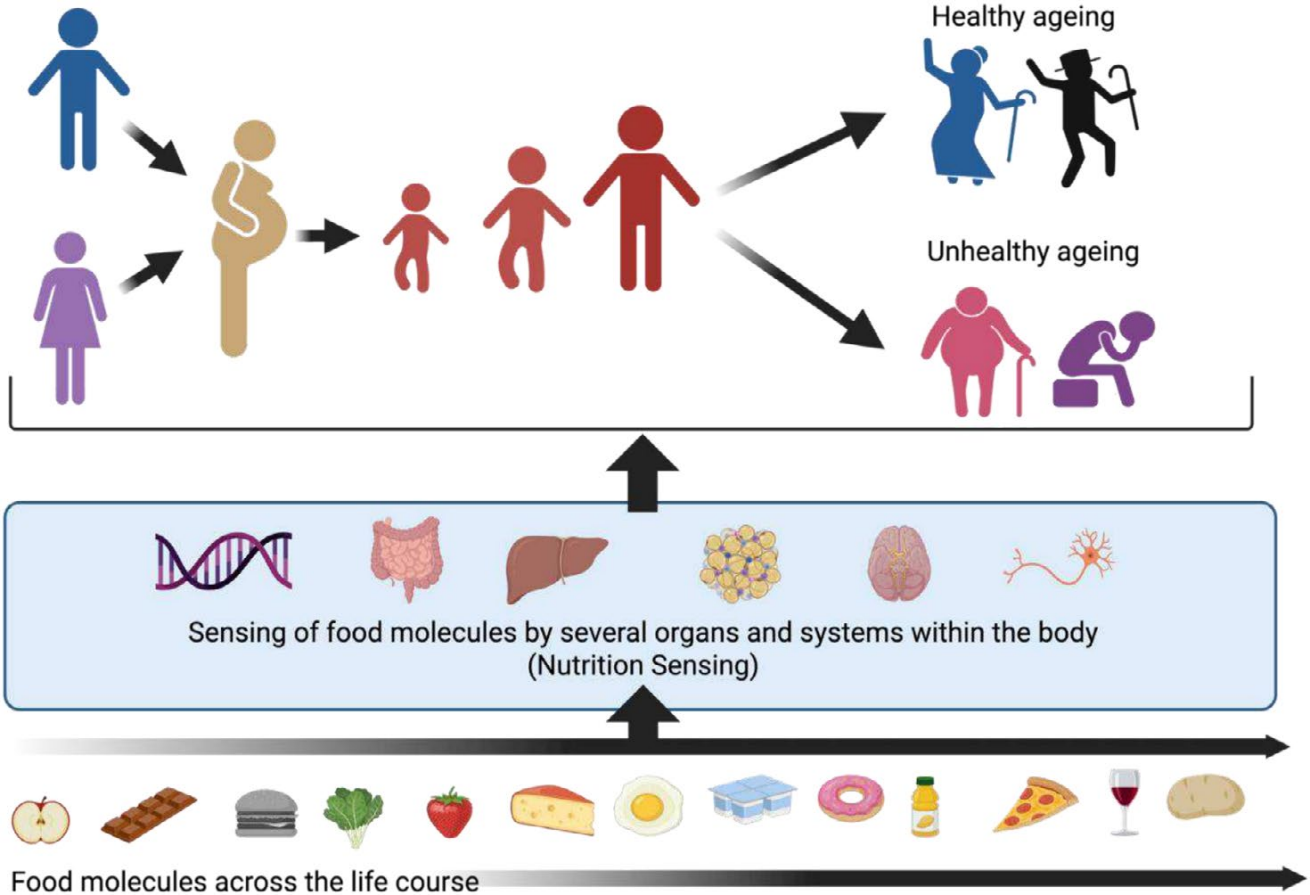
Potential impact of nutrition sensing on ageing across all stages of life.

Based on such evidence, we hypothesise that the body's ability to sense and respond to nutrients plays a pivotal role in the ageing process throughout life (Figure 1). However, there is still much to learn about nutrient‐body interactions in humans and how this affects ageing, at multiple scales, from the nutrient‐induced molecular level signalling to the food environment. Through our Ageing and Nutrition Sensing (AGENTS) Network, we aim to advance the understanding of how nutrition sensing systems change with advancing years and how this influences the ageing process and, consequently, the risk of common age‐related diseases. A deeper understanding will inform approaches and interventions to promote healthy ageing, leading to enhancements in lifespan and health span for our ageing population.

## 2 Our Network

The AGEing and Nutrition Sensing (AGENTS) Network is comprised of a multi‐disciplinary consortium of leading researchers and early career researchers (ECRs) with expertise across ageing, cell biology, nutrition, epidemiology and policy. The AGENTS Network was awarded a UK Research and Innovation‐Biotechnology and Biological Sciences Research Council (UKRI‐BBSRC) Ageing Network grant in 2022. The network aims to develop a programme of research that will lead to an increase in healthy life years. Our work aims to identify and address the critical unanswered questions relating to ageing and nutrition sensing. The following document represents the consensus opinion of the AGENTS Network concerning the key research challenges to address regarding the nutrition sensing factors that contribute to the decline in health during ageing and that increase the burden of age‐related disability and disease. In addition, we provide an overview of the AGENTS Network and how it is building a community to synthesise new knowledge around ageing and nutrition sensing that can be used to inform policy and practice for societal impact.

## 3 Nutrition Sensing

### 3.1 Definition and Current Evidence

Our network has defined nutrition sensing as:

Systems at multiple scales that detect and respond to nutritional flux.

In addition, we have adopted the WHO definition of biological ageing:

The impact of the accumulation of a wide variety of molecular and cellular damage over time. This leads to a gradual decrease in physical and mental capacity, a growing risk of disease and ultimately death. These changes are neither linear nor consistent. (WHO 2024)

Nutrition is sensed by the human body via a complex system consisting of a range of nutritional receptors, including G protein‐coupled receptors (GPCRs), ion channels and their associated downstream signalling pathways (Efeyan et al. 2015). Insulin, Insulin‐like Growth Factor‐1 (IGF‐1), mechanistic Target of Rapamycin (mTOR), AMP‐Activated Protein Kinase (AMPK) and sirtuin pathways are examples of nutrient sensing pathways that have been shown to impact ageing (Lee and Lee 2022; Salminen and Kaarniranta 2012; Robida‐Stubbs et al. 2012; Kanfi et al. 2012). For example, deregulation of insulin sensitivity becomes more common during ageing (i.e., insulin resistance) which reduces the body's capacity to control blood glucose concentration (Chang and Halter 2003). This can increase inflammation, oxidative stress and liver fat accumulation, subsequently increasing the risk of diseases such as diabetes, atherosclerosis and fatty liver disease (Utzschneider and Kahn 2006; Reaven 2012; Marott et al. 2019; Hurrle and Hsu 2017). Accordingly, centenarians were found to have high insulin sensitivity and low circulating concentrations of insulin (Barbieri et al. 2008).

As previously mentioned, caloric restriction increases lifespan in multiple model organisms including mammals such as rodents (Fontana 2006). Insulin, IGF‐1 and mTORC1 signal the presence of nutrition whereas AMPK and sirtuins signal the lack of nutrition. Accordingly, downregulation of insulin, IGF‐1 and mTORC1 (Kaeberlein et al. 2005; Lee and Lee 2022) but the upregulation of the AMPK and sirtuin pathway (Stancu 2015; Mouchiroud et al. 2013), signalling caloric restriction, were shown to increase lifespan in model organisms. Epidemiological evidence from Japanese (Okinawans) centenarians indicates that mild calorie restriction (17%) may enhance lifespan in humans although clinical evidence is currently lacking in this area (Willcox et al. 2007).

On the contrary to the above discussion, more extreme restriction of nutritional intake resulting in malnutrition was observed to increase frailty, morbidity, and mortality in older adults (Söderström et al. 2017; Coelho‐Júnior et al. 2018). This is specifically prominent in older adults suffering from anorexia of ageing, which is the loss of appetite and low food intake observed in some older adults (Landi et al. 2016). Another mechanism by which nutrition is acutely sensed is the release of appetite‐reducing hormones such as Glucagon‐like peptide 1 (GLP‐1) and Peptide Tyrosine Tyrosine (PYY) (De Silva and Bloom 2012). While several nutrition and metabolite‐sensing GPCRs are known to drive the release of GLP‐1 and PYY (Husted et al. 2017), how these receptor mechanisms change in activity with age is poorly understood. However, our team recently demonstrated that the postprandial release of appetite‐reducing hormones is enhanced in older adults with anorexia of ageing (Dagbasi, Warner, et al. 2024). This may indicate hypersensitivity in nutrition‐sensing mechanisms linking to the release of these hormones. Recent evidence also highlights the correlation of biological age as assessed by DNA methylation (Epigenetic clock/GrimAge) and appetite reduction in older adults, indicating the potential role of epigenetic factors in nutrition‐sensing pathways (Turesson et al. 2025). The role of nutrition sensing in the anorexia of ageing was reviewed in detail elsewhere (Dagbasi, Fuller, et al. 2024).

Good nutrition has other benefits during ageing beyond merely extended lifespan. For example, adherence to a Mediterranean diet has been linked to better cognitive ability during ageing (Turesson et al. 2025) and has been shown to be associated with longer telomere length in women (García‐Calzón et al. 2016), shortening of which is associated with ageing (Blackburn et al. 2006; Calado and Young 2009). The characteristic foods and nutrients in a Mediterranean diet have benefits for at least nine hallmarks of ageing (Shannon et al. 2021).

The food environment is a factor in nutrition sensing that also impacts ageing. For example, loneliness and social isolation increase the risk of malnutrition in older adults due to multiple factors such as depression, inability to cook or reduced motivation to cook/eat (Steptoe et al. 2024; Boulos et al. 2017). Socioeconomic status is an important determinant of ageing, with individuals from lower socioeconomic backgrounds showing accelerated ageing and increased risk of morbidity/mortality (Steptoe and Zaninotto 2020). Food insecurity may be one contributing factor (Leroux et al. 2020) which will be discussed further in our Project 1 section below.

Overall, current evidence suggests that from molecular mechanisms to the food environment, nutrition sensing impacts the ageing trajectory although the evidence in humans remains limited.

## 4 Challenge Areas Around Nutrition Sensing

The AGENTS Network identified the following areas as challenges to advance understanding of the field of ageing and nutrition sensing:

1. Many common non‐communicable diseases result from dysregulation of nutrition sensing pathways, which are poorly understood.
2. Evidence suggests that nutrition sensing plays an important role in homeostatic mechanisms from the cell to whole body metabolism and food choice, but these mechanisms remain understudied, especially in humans.
3. There is a need to understand how an individual's environment influences nutrition sensing and vice versa.
4. There are well described fundamental biological nutrition sensing pathways that influence lifespan in unicellular organisms and small mammals, yet understanding of how such pathways influence whole body human physiology is limited.
5. There is a lack of established and accepted biomarkers for mapping the links between nutrition sensing and human ageing.

To advance the understanding in these areas, the AGENTS Network has developed (and is supporting, via pump priming funds) four multidisciplinary projects. These projects bring together researchers from multiple institutions and disciplines to stimulate new research thinking and innovative research approaches with the aim of generating larger research projects that will bring new understanding to the field. Each project also has an ECR lead to support the development of ECRs by providing leadership opportunities. In addition to the projects, we developed an ECR network. These projects are detailed below and the ECR network is detailed in Section 5.

### 4.1 Project 1: Understanding and Influencing the Mechanisms of Ageing Through Dietary Interventions

#### 4.1.1 Addressing Challenge Areas: 2, 3 and 5

##### 4.1.1.1 Context

Ageing is a multifactorial process, associated with reduced function and increased risk of morbidity and mortality (PHE 2018). A range of biomarkers of ageing have been proposed which may be valuable for predicting future health and survival, and monitoring responses to dietary, lifestyle or pharmacological interventions (Moqri et al. 2023; Belsky et al. 2022; Bortz et al. 2023; Martin‐Ruiz et al. 2011; Bürkle et al. 2015; Lara et al. 2015, 2013). Research is needed to identify the most appropriate biomarkers of ageing and to explore how these biomarkers are impacted by dietary intervention.

##### 4.1.1.2 Aim

This project aims to (a) identify tractable biomarkers of ageing which can serve as outcome measures in future intervention studies, and (b) explore the impact of dietary interventions on these age‐related biomarkers.

##### 4.1.1.3 Approach

A modified Delphi study will be conducted to generate a global expert consensus on appropriate biomarkers for ageing for use as outcome measures in intervention studies. Over three iterative rounds, an expert panel comprising ~50 international experts will identify biomarkers of ageing and appraise their suitability for use in different research settings. The Delphi stage of the study will be funded by the network. The second stage will investigate the effect of manipulations in meal timing on a selection of biomarkers of ageing identified via the Delphi study. This will include mimicking the irregular, unpredictable meal timing experienced by individuals living with food insecurity (hypothesised to accelerate the ageing trajectory (Andrews et al. 2021)) and a more regular meal timing manipulation (time restricted eating, hypothesised to slow the ageing trajectory (Serra et al. 2019)). The study will provide a better understanding of the social and dietary factors impacting the ageing process while unravelling aspects of the mechanisms behind it. Funding for this project will be sought from another source but will be strengthened by AGENTS funding and preliminary data collection.

##### 4.1.1.4 Current Progress

This team has completed and published a Delphi study on the biomarkers of ageing, including 116 international panel members (Perri et al. 2025). They have successfully completed a pilot study (*n* = 12) of their meal timing manipulation and are currently planning additional human studies.

### 4.2 Project 2: Gene Polymorphisms and Mechanistic Understanding of the Impact of Nutrition on Healthy Ageing

#### 4.2.1 Addressing Challenge Areas: 1, 2 and 4

##### 4.2.1.1 Context

Nutrition is an important factor that impacts the ageing process and the risk of morbidity (Shannon et al. 2021). However, response to nutrition differs greatly between people (De Lucia et al. 2020). We hypothesise that this heterogeneity in response to dietary factors is due to polymorphisms in nutrition sensing genes.

##### 4.2.1.2 Aim

To understand the impact of nutrition, genetic polymorphisms, and gene expression on healthy ageing.

##### 4.2.1.3 Approach

In the first stage, which is entirely funded and supported by the AGENTS Network, we will identify candidate nutrient and nutrition sensing genes based on existing literature. We will then use available platforms (e.g., GTEX) to identify single nucleotide polymorphisms (SNPs) that act as expression quantitative trait loci (eQTLs) for the candidate genes. Phenome‐wide association study (PheWas) analysis will then be carried out on all identified SNPs to identify traits of interest. Focus will be on SNPs linked to nutrition, diet, ageing and metabolism to select independent SNPs that are associated with phenotypes of interest. During this stage, we will also optimise a novel pipeline that automates the above process and allows for more efficient and unbiased analysis.

During the second stage, the network will be instrumental in supporting the development of a 3‐year grant proposal. As part of this proposal, we will build on pilot and proof‐of‐concept data collected in stage 1 to delineate the effect of the selected candidate SNPs on ageing, frailty and sarcopenia phenotypes (e.g., BMI, body composition, cognition and muscle strength and walking pace). We will also assess whether these associations are mediated or moderated by diet. Using causal inference methods, such as Mendelian Randomization, we will finally investigate if there is a causal relationship between candidate gene expression and ageing phenotypes. This stage will use large cohort data such as the UK Biobank and TwinsUK cohorts. These predicted outcomes will not only advance scientific understanding but also have practical applications in promoting healthier ageing through personalised interventions, with the potential to inform public health policies.

The third and final stage of this project will involve mechanistic investigation of nutrition sensing pathways using in vitro and in vivo models to determine the impact of selected polymorphisms on gastrointestinal and neuronal function. In this stage we will also explore the potential of a personalised human interventional trial. Funding for this project will be sought from other sources but will be strengthened by AGENTS funding, collaborations, and preliminary data collection.

#### 4.2.2 Current Progress

This project developed a novel pipeline to investigate the effect of SNPs in nutrition sensing genes on dietary intake and general health. A paper on this pipeline is being prepared for publication.

### 4.3 Project 3: Understanding the Biological Mechanism of Anorexia of Ageing

#### 4.3.1 Addressing Challenge Areas: 1–4

##### 4.3.1.1 Context

Around one in three community‐dwelling older adults are at risk of undernutrition due to an age‐related reduction in appetite, termed anorexia of ageing (AA) (Landi et al. 2016). As insufficient nutrient and energy intake is associated with sarcopenia (Sieber 2019), morbidity and mortality (Söderström et al. 2017) in later life, ensuring older adults retain a good appetite is vital for healthy ageing.

##### 4.3.1.2 Aim

To enhance our understanding of the biological mechanism underpinning AA. While the control of appetite is multifactorial, with hedonic and homeostatic inputs (Lutter and Nestler 2009), we focus on nutrition‐sensing mechanisms. There is evidence for age‐related changes in appetite‐associated gut hormone response to feeding, suggesting alterations in how the gut senses nutrient delivery to the gastrointestinal tract (GIT).

##### 4.3.1.3 Approach

The first objective is to develop a valid means of phenotyping older adults with AA. This will allow us to explore changes in nutrition sensing processes which are specific to those with AA—not merely a function of ageing—and hence may be causal of appetite decline. The second objective is to characterise gut hormone responses to feeding in older adults with AA and those with a healthy appetite. This will identify which nutrition sensing processes appear dysregulated in AA. The third objective is to determine mechanisms which may explain dysregulation in nutrition sensing, such as changes in the gut environment and molecular changes in nutrition sensing and signalling pathways. This will be achieved by examining gastrointestinal tissue from older adults and experimental studies using organoid models.

##### 4.3.1.4 Current Progress

The project resulted in the publication of two review articles: one on the potential mechanisms of the anorexia of ageing (Dagbasi, Fuller, et al. 2024) and the other on the appetite regulating gut hormones through life stages (Holliday et al. 2025). The collaborations within this project also resulted in a research article showing differences in circulating concentrations of gut hormones in older adults compared with young adults which are proposed to impact appetite (Dagbasi, Warner, et al. 2024).

### 4.4 Project 4: Exploring the Impact of Protein Quality and Dietary Fibre on Gut Barrier Function, Inflammation and Body Composition

#### 4.4.1 Addressing Challenge Areas: 1–5

##### 4.4.1.1 Context

A decline in GIT barrier function is associated with inflammation, which has a significant negative effect on muscle mass and function (Westbury et al. 2018). A decline in GIT barrier function and low‐grade inflammation are associated with ageing in animal models, but robust data in humans is lacking (Chung et al. 2009). Maintaining sufficient intake of high‐quality protein is also essential for maintaining muscle mass and function in ageing, though robust data on protein requirements associated with the ageing process are scarce (Deer and Volpi 2015). Dietary protein and dietary fibre have been demonstrated to have an impact on GIT integrity and optimising gut health, which may be an important modifiable aspect of the ageing process (Prokopidis et al. 2020; Shivakoti et al. 2022). Therefore, replacing animal sources of protein with sustainable plant proteins may have many benefits in ageing, but studies on protein quality are lacking.

##### 4.4.1.2 Aims

To determine the impact of protein quality and dietary fibre on gut barrier function, inflammation, body composition and muscle function in older adults.

##### 4.4.1.3 Approach

The project will have three stages. This project firstly aims to collaborate with industry partners to develop biomarkers of biological age and gut barrier function. Secondly, it aims to engage with the public to develop an understanding of acceptability of changes to protein sources (e.g., legume vs. animal‐based protein). Thirdly, it aims to bring together preliminary data with public and stakeholder engagement to co‐create an intervention study to test the effect of alternative sustainable protein sources and increased dietary fibre on gut health, inflammation and age‐related health outcomes.

##### 4.4.1.4 Current progress

This team has produced a systematic review on the effect of sustainable proteins on gut health in older adults (Jones et al. 2024). The project also resulted in two research papers. One of these is showing associations between dietary intake, inflammation and gut function in older adults (Jones et al. 2025). The other paper is showing that high fibre and plant protein intakes are associated with lower inflammation in older adults (Jain et al. 2025).

## 5 Research Community

**FIGURE 2:**
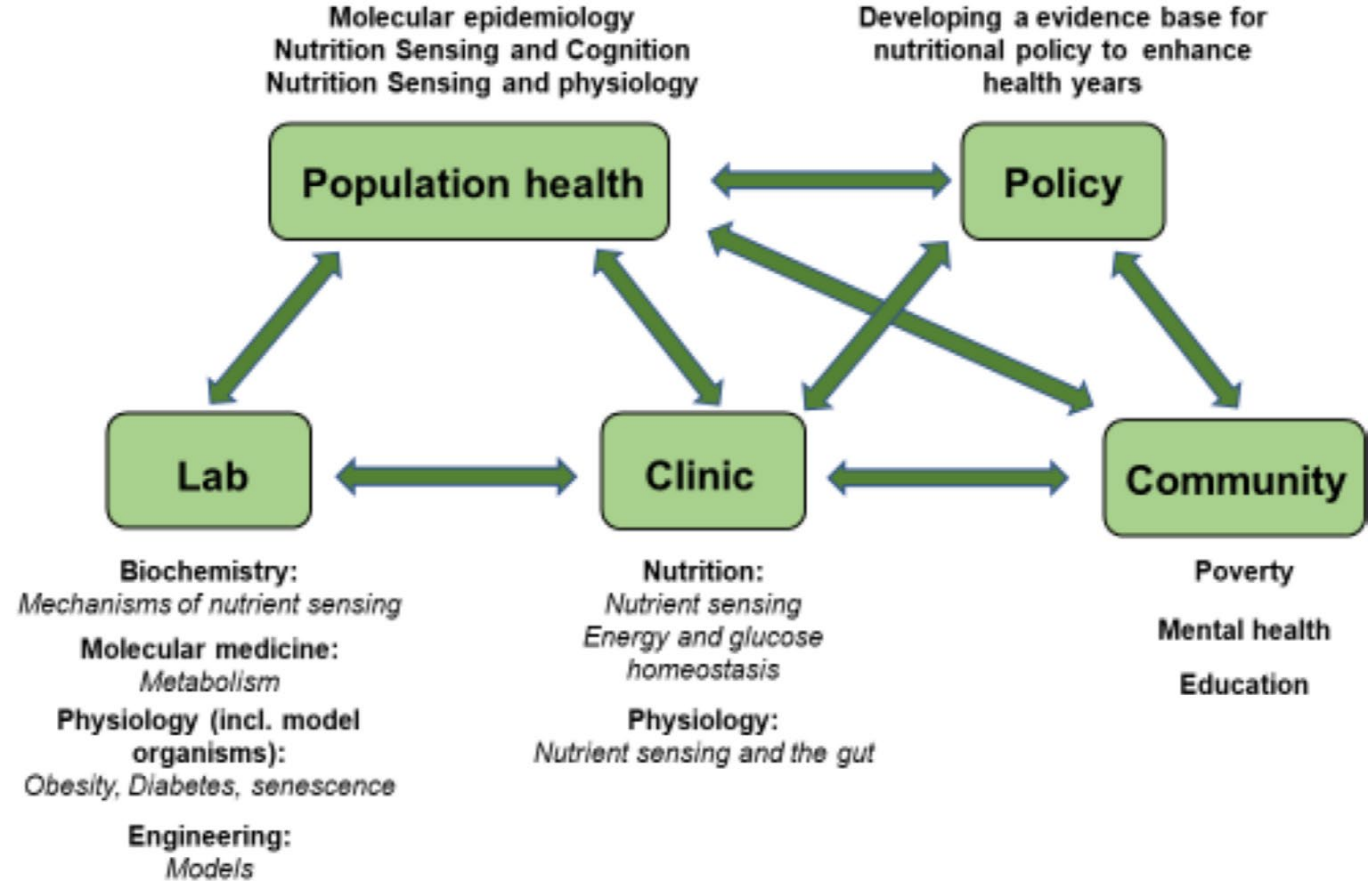
The interdisciplinary working plan by AGENTs Network.

Each project embraces interdisciplinary research and adopts a team science approach, as highlighted in Figure 2 below, to build holistic understanding of the research.

The AGENTS Network is committed to nurturing and propelling the next generation of scientists in the field. The formulation of each project team not only strategically considered the blending of expertise and the promotion of interdisciplinarity, but also ensured a diversity of experienced senior research group leaders, mid‐career academics who are developing research independence and leadership, and ECRs. We have initiated the informal mentoring of mid‐career academics and ECRs by senior PIs within each project, with mid‐career academics and ECRs afforded the opportunity to embrace leadership roles, including applying for research funding. An ECR development programme (ECR network) has been established that includes initiatives such as workshops on academic writing and presenting research, networking opportunities, and Q&A sessions for guidance on career pathways. The ECR network provided pump‐priming funds to 26 ECRs for smaller projects in the field of ageing and nutrition sensing, for placements, for conference attendances and for patient and public involvement and engagement (PPIE) events. The network was awarded Flexible Talent Mobility Accounts by BBSRC which funded 11 ECRs for placements within the UK and also international placements.

## 6 Engaging Stakeholders for Research Impact, Public Engagement, and Informing Policy

At the point of the network's conception, our membership consisted primarily of academics. However, we are working to broaden our membership to include key stakeholders from industry, health and social care, community groups, charities and the social sciences.

As a network, we are passionate about research with societal purpose and impact. We aim to establish relationships with relevant community groups and organisations (such as the Elders Council of Newcastle and Age UK) to forge pathways to impact and foster public engagement and involvement. We hope to engage representatives from such organisations in the network, ensuring the voice of older adults helps shape our research agenda moving forward.

An overarching long‐term aim of all projects is to bring scientific evidence to influence and inform policy that will improve the health and wellbeing of older people. Our research aligns with recent strategic priorities to tackle malnutrition as identified by the James Lind Alliance (JLA 2025; Jones et al. 2020), and with the priorities outlined in the Scientific Advisory Committee on Nutrition's position statement on nutrition and older adults living in the community (Van Dijk et al. 2021). Nutrition sensing affects a wide variety of fundamental physiological systems that determine both homeostatic mechanisms and physical function. We believe that changes in nutrition sensing systems can have a profound effect later in life. It must be remembered what we mean by nutrition sensing; this is not just fundamental cellular processes but also social aspects such as isolation and loneliness. Factors such as the loss of sight and taste can also contribute to a reduced capacity to sense the nutrition/nutrient environment. Such issues often coexist. At the present time, the consequences of changes in nutrition sensing such as sarcopenia or malnutrition form part of policy documents. The drive to understand the fundamental cellular mechanisms behind these consequences has not followed. Understanding how the fundamental biology interacts with social aspects of life could lead to preventative policies that could reduce morbidity and mortality in the older population.

### 6.1 Current Progress

Our membership grew from 15 members in February 2022 to 124 members in May 2025. The growth in the network over the years has been largely due to networking through a number of channels such as social media and scientific meetings. We held a PPIE meeting with Elders Council in Newcastle and collaborated with industry partner Glycanage. Our biggest challenge has been engagement beyond biological sciences. At the outset, we want to bring in social science expertise into the network. We had a number of social scientists attending our events to give talks; however, we failed to attract expertise to our projects. The reason why we were unable to do this is still unclear and we are still working to attract social scientists to our network.

## 7 Summary

By building a new multi‐disciplinary, multi‐institutional research community, and adopting a team science approach, the AGENTS Network will deliver new biological understanding to the field of ageing and nutrition sensing. The AGENTS Network believes that to advance understanding of human ageing and health span, it is essential to pursue investigations at many scales from single cell biology up to population science. It is essential that this embraces interdisciplinary collaboration across biological and social science and includes the role of industry and civil society.

## Conflicts of Interest

Gary Frost is the director of Melico Sciences and has received funding from Nestle, Quorn and Sosei Heptares.

## Data Availability

The authors have nothing to report.
